# In situ carbon dioxide capture to co-produce 1,3-propanediol, biohydrogen and micro-nano calcium carbonate from crude glycerol by *Clostridium butyricum*

**DOI:** 10.1186/s13068-022-02190-2

**Published:** 2022-09-03

**Authors:** Xiao-Li Wang, Jin-Jie Zhou, Sheng Liu, Ya-Qin Sun, Zhi-Long Xiu

**Affiliations:** School of Bioengineering, Dalian University of Technology, No. 2 Linggong Road, Ganjingzi District, Dalian, 116024 Liaoning People’s Republic of China

**Keywords:** 1,3-Propanediol, Micro-nano-CaCO_3_, Green hydrogen, CO_2_ capture, Waste glycerol

## Abstract

**Background:**

Climate change caused by greenhouse gas emission has become a global hot topic. Although biotechnology is considered as an environmentally friendly method to produce chemicals, almost all biochemicals face carbon dioxide emission from inevitable respiration and energy metabolism of most microorganisms. To cater for the broad prospect of biochemicals, bioprocess optimization of diverse valuable products is becoming increasingly important for environmental sustainability and cleaner production. Based on Ca(OH)_2_ as a CO_2_ capture agent and pH regulator, a bioprocess was proposed for co-production of 1,3-propanediol (1,3-PDO), biohydrogen and micro-nano CaCO_3_ by *Clostridium butyricum* DL07.

**Results:**

In fed-batch fermentation, the maximum concentration of 1,3-PDO reached up to 88.6 g/L with an overall productivity of 5.54 g/L/h. This productivity is 31.9% higher than the highest value previously reports (4.20 g/L/h). In addition, the ratio of H_2_ to CO_2_ in exhaust gas showed a remarkable 152-fold increase in the 5 M Ca(OH)_2_ group compared to 5 M NaOH as the CO_2_ capture agent. Green hydrogen in exhaust gas ranged between 17.2% and 20.2%, with the remainder being N_2_ with negligible CO_2_ emissions. During CO_2_ capture in situ, micro-nano calcite particles of CaCO_3_ with sizes in the range of 300 nm to 20 µm were formed simultaneously. Moreover, when compared with 5M NaOH group, the concentrations of soluble salts and proteins in the fermentation broth of 5 M Ca(OH)_2_ group were notably reduced by 53.6% and 44.1%, respectively. The remarkable reduction of soluble salts and proteins would contribute to the separation of 1,3-PDO.

**Conclusions:**

Ca(OH)_2_ was used as a CO_2_ capture agent and pH regulator in this study to promote the production of 1,3-PDO. Meanwhile, micro-nano CaCO_3_ and green H_2_ were co-produced. In addition, the soluble salts and proteins in the fermentation broth were significantly reduced.

**Graphical Abstract:**

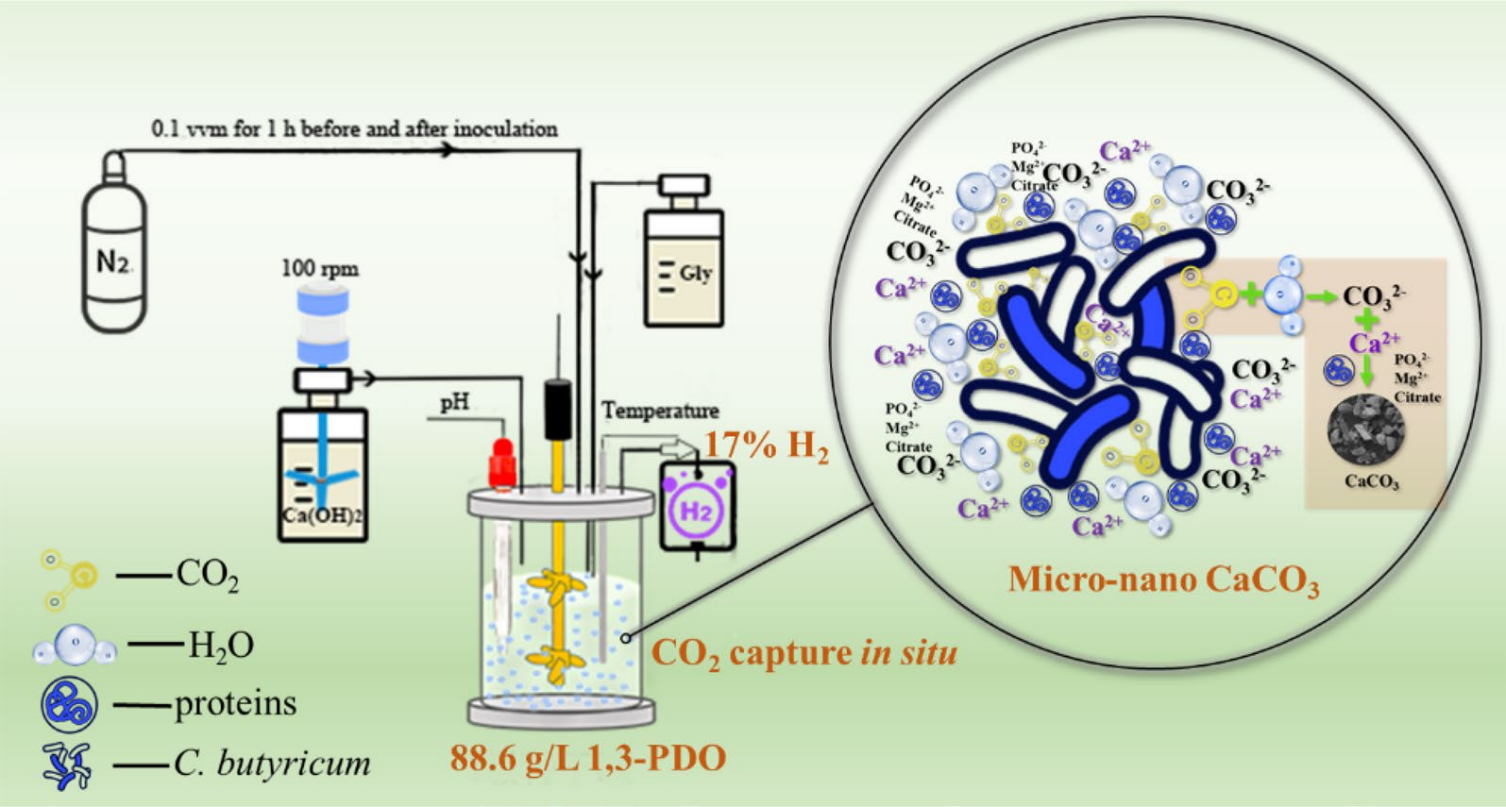

**Supplementary Information:**

The online version contains supplementary material available at 10.1186/s13068-022-02190-2.

## Background

There is a global consensus on the urgent need to reduce carbon footprints to develop a renewable economy and protect our habitable planet. For more than 40,000 years prior to 1912, the atmospheric CO_2_ concentration was no higher than 300 ppm, but has risen at an astonishing rate over the last 100 years, reaching 417 ppm by July 2021 [1, 2]. It is estimated that the level of atmospheric CO_2_ will up to 500 ppm by 2045, which may pose the most severe threat to the environment and public health [3]. A growing number of countries have declared that they will reduce CO_2_ emissions to net-zero within decades, which has greatly encouraged the ambition to tackle climate change [4]. In this case, biotechnology is favored by various countries, which is known as an environmentally friendly method to convert renewable resources to chemicals in a mild reaction. Therefore, biochemicals such as bioethanol, lactic acid, 1,3-PDO, and so on were produced increasingly year by year. However, when considering techno-economic analysis (TEA) of a process, almost all biochemicals production generates greenhouse gas according to their life cycle assessment (LCA)) because of CO_2_ from inevitable respiration and energy metabolism of most microorganisms [5]. In detail, the commercial-scale annual production of some biochemicals and the corresponding CO_2_ production are presented in Table 1, which are calculated according to the reported microbial metabolic network and the stoichiometry of the reactions. As a result, the annual emission of CO_2_ from biochemicals is at least 84.4 million tons, which should be avoided in the future to achieve carbon neutrality. More recently, some proposals for biochemicals production by researchers have focused on optimizing processes for environmental sustainability, identifying cost-effective [12], and new methods (e.g., 3G biorefineries) to capture and convert CO_2_ into valuable products [3]. To cater for the broad prospect of biochemicals, the industrially implementable process of integrating waste organic carbon utilization, diversifying valuable products, cleaning production, and recycling CO_2_ is becoming essential.

**Table 1:** Production of some industrial biochemicals by microbial fermentation in major relevant countries and induced CO_2_ production in the fermentation

Biochemicals	Production capacity (tons/a year)	Major production Country	induced CO_2_ production (tons/a year)	References
Bioethanol	88 million	USA, Brazil &China	84.2 million	[6, 7]
1,3-Propanediol	63,000	USA	118,030	[7, 8]
1,4-Butanediol	105,000	USA, Germany & Italy	13,310	[7, 9]
Butanol	21,460	China	25,480	[10, 11]

Crude glycerol is a primary by-product of the oleochemical industry, such as biodiesel, soap, fatty acid, and fatty ester production [13]. For every 100 tons of biodiesel produced via transesterification, 10 tons of glycerol are produced as the by-product [14, 15]. What is more, glycerol is also produced in the process of bioethanol production, accounting for 7–8% (w/w) of bioethanol [16]. With the mass production of biodiesel and bioethanol, the surplus of crude glycerol results in a low price of pure glycerol, although glycerol is widely used as a chemical in cosmetics, food, solvent, and pharmaceutical industries. For that reason, crude glycerol is considered as an industrial waste production not only because of its low price but also its pollution to the environment [17]. Thus, the conversion of waste glycerol to high value-added products by microbial fermentation is attracting much attention considering the use of a renewable feedstock and environmental performance [18, 19].

Glycerol, as a substrate, could be converted into many value-added chemicals, such as 1,3-propanediol (1,3-PDO), 2,3-butanediol, citric acid, n-butanol, ethanol, lipids, docosahexaenoic acid (DHA), and eicosapentaenoic acid (EPA), using a variety of microorganisms via different metabolic pathways [13, 20–26]. Among these products, 1,3-PDO, an important bulk chemical, is one of the most valuable products. It has been extensively applied in cosmetics, pharmaceuticals, and solvent industries and is especially used for the synthesis of polyester materials, such as polytrimethylene terephthalate (PTT) and polytrimethylene ether glycol [27]. The demand for 1,3-PDO has increased sharply, because PTT has a wide range of applications in the textile industry due to its excellent properties, such as stain resistance, good softness, and low temperature dyeing [28, 29]. The production of 1,3-PDO from glycerol has been extensively investigated using anaerobic or micro-aerobic fermentation with various bacteria, such as *Klebsiella pneumoniae*, *Clostridium butyricum* and *Lactobacillus reuteri* and so on [30–32]. *C. butyricum* is considered as one of the most excellent 1,3-PDO producers due to its superior performance in efficient 1,3-PDO production and less by-products. Meanwhile, it is suitable for industrial production of 1,3-PDO, because it is a probiotic in the intestinal tract and B_12_-independent bacterium [33]. In the previous report, *C. butyricum* DL07 could produce 104.8 g/L 1,3-PDO from glycerol, and the productivity was up to 3.38 g/L/h in fed-batch fermentation [34], which are among the best results in natural bacteria.

The intracellular metabolic pathway of glycerol in *C. butyricum* has been well-recognized by intracellular metabolic analysis [25]. 1,3-PDO is produced via the metabolic reduction pathway, accompanied by the formation of organic acids, such as butyric acid, acetic acid, and lactic acid in the oxidation branch [35]. Usually, alkaline pH regulators are required to neutralize organic acids and control a constant pH during the fermentation of *C*. *butyricum*. Otherwise, the organic acids could inhibit 1,3-PDO production, because more energy is needed to maintain the pH in the cell [36, 37]. In general, soluble alkalis such as NaOH, KOH, and ammonia are used to maintain pH (7.0) during fermentation for 1,3-PDO production [32, 38, 39]. However, as the addition of alkalis, the cations such as Na^+^, K^+^ and NH_4_^+^ from the alkalis are introduced with high concentration into the fermentation broth, which cause high osmotic pressure and inhibiting the growth of bacterial cells [40]. Furthermore, CO_2_ and H_2_ are produced in the oxidation branch of glycerol metabolism [41]. The formation of CO_2_ not only puts pressure on the environment but also reduces carbon utilization. In addition, the dissolution of CO_2_ in fermentation broth would require more alkaline solution to neutralize the fermentation pH. Then, the large amounts of soluble salt in the fermentation broth bring difficulty in the separation of 1,3-PDO. Therefore, the recovery of CO_2_ and the reduction of soluble salt concentration in fermentation broth are expected in the microbial production and separation of 1,3-PDO. At the same time, H_2_ with a relatively high purity will be available as a clean, sustainable, and ideal energy resource.

Nano- and micro-particles of calcium carbonate (CaCO_3_), an important material, have been widely used in many fields, such as the construction industry, paper industry, cosmetics, toothpastes, water treatment, pigments, and drug delivery systems [42, 43]. The synthesis of CaCO_3_ has attracted many researchers’ interests in recent years because of its good properties, such as the high ratio of surface area to volume, high porosity, non-toxicity and compatibility toward the human body [44]. CaCO_3_ can exist in three crystal forms: calcite, vaterite, and aragonite. Calcite is the most stable polymorph and one of the most common minerals on Earth, serving as the main component of sedimentary limestone. At present, calcite (CaCO_3_) could be synthesized by chemical method and microbially induced precipitation (MIP). MIP is more favored by researchers over chemical synthesis, because it can obtain the controlled single crystal [45]. Regrettably, bacteria-induced CaCO_3_ usually takes several days and is only produced in small quantities. In a report, *Rhodococcus degradans* BaTD-248 was cultured for 3 and 21 days to produce CaCO_3_ [46]. Some ions, such as magnesium, phosphate, citrate, and silicate, as well as the incorporation of amino acid, proteins, extracellular polymeric substance (EPS), and other macromolecules, usually affect bacteria-induced CaCO_3_ [45–48].

The aim of this study is to propose a novel integrated process for the production of multiple products while avoiding CO_2_ emission during fermentation. The optimized process could achieve high-efficient production of 1,3-PDO, high ratio H_2_ and micro-nano CaCO_3_ from industrial waste glycerol by *C. butyricum* DL07. In fed-batch fermentations, NaOH, Ca(OH)_2_ and double CO_2_ capture agents (NaOH & Ca(OH)_2_) will act as CO_2_ capture agents and pH regulators for 1,3-PDO production. The various Ca(OH)_2_ concentrations and stirring speeds were investigated to produce 1,3-PDO, H_2_, and micro-nano CaCO_3_. Moreover, the effects of this process on the salt concentration, ionic composition and soluble protein concentration in the fermentation broth were discussed.

## Results and discussion

### Effects of carbon dioxide capture agents on 1,3-PDO production

#### Efficient 1,3-PDO production using NaOH as a CO_2_ capture agent and pH regulator

**Fig. 1:**
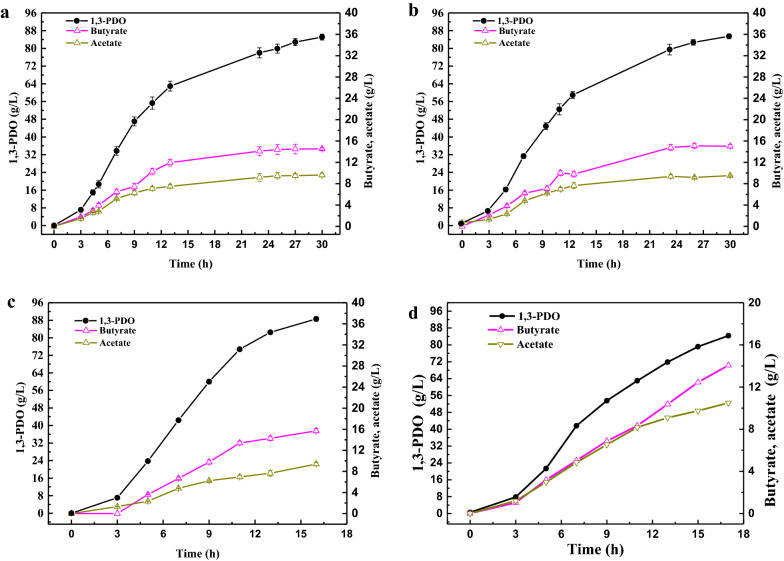
Profit of products using NaOH (**a**, **b**) or/and Ca(OH)_2_ (**c**, **d**) as the CO_2_ capture agent and pH regulator. 5 M NaOH was employed in **a** and **b**, where **a** was flushed with nitrogen gas for 1 h before and after inoculation and **b** with continuous nitrogen gas flushing throughout the fermentation; 5 M Ca(OH)_2_ suspension was used in under similar nitrogen gas flushing conditions with **a**; 5 M NaOH and 5 M Ca(OH)_2_ suspension were used in **d** under similar nitrogen gas flushing conditions with **a**

During the microbial fermentation of 1,3-PDO, organic acids were produced as by-products, as well as CO_2_ as exhaust gas, due to redox homeostasis and energy balance during the glycerol metabolism [25, 49, 50]. As a CO_2_ capture agent and pH regulator, NaOH was selected to absorb CO_2_ and neutralize the organic acids, e.g., 5 M NaOH solution used for anaerobic fermentations by *C. butyricum* DL07. To create anaerobic fermentation environment, nitrogen gas was bubbled into the fermentation medium for 1 h before and after inoculation, respectively, or continuously throughout the whole fermentation process. Regardless of the conditions in the preceding two cases, efficient 1,3-PDO production could be achieved, as shown in Fig. 1a, b. A high 1,3-PDO concentration of 85.1 g/L was obtained with a satisfying yield of 0.504 g 1,3-PDO/g glycerol and a productivity of 2.84 g/L/h when nitrogen gas was introduced for 1 h before and after inoculation, respectively. Moreover, the concentrations of butyric acid and acetic acid were 14.5 and 9.63 g/L, respectively. While nitrogen gas was continuously pumped into the bioreactor, 1,3-PDO concentration achieved 85.5 g/L with almost the same yield of 0.506 g 1,3-PDO /g glycerol and productivity of 2.85 g/L/h, as well as the concentrations of butyric acid and acetic acid (15.0 and 9.53 g/L, respectively). Fermentations in both of the preceding cases took the same time of 30 h to complete fermentations. Clearly, there was little difference in 1,3-PDO and organic acids production in the above two cases. However, the consumption of 5 M NaOH solution showed significant differences The consumption of 5M NaOH solution was 328 g in the first case, whereas only 241 g 5 M NaOH in the second. This indicates that there were significant differences in the amount of CO_2_ captured in the two cases.

*C. butyricum* IK 124 produced 80.1 g/L of 1,3-PDO with a productivity of 1.80 g/L/h in a fed-batch fermentation using crude glycerol as the substrate [51]. Similar results were obtained, i.e., 80.2 g/L of 1,3-PDO and 1.16 g/L/h of productivity, using *K. pneumoniae* DSM 4799 from crude glycerol [38]. In contrast to previous reported 1,3-PDO production from crude glycerol, *C. butyricum* DL07 could achieve higher concentration (85.0 g/L 1,3-PDO) and productivity (2.84 or 2.85 g/L/h) in this study. When the above two flushing nitrogen gas modes were compared, they had almost no effect on the production of 1,3-PDO and by-products. About 0.340 mol/L organic acids were generated in both fermentation groups. In other words, about 216 g 5 M NaOH solution was required to neutralize the organic acids produced during fermentation, theoretically. However, the theoretical value was less than the actual consumption of 5 M NaOH solution in the first case (328 g) or the second one (241 g), implying that CO_2_ fixation levels differed, i.e., 0.24 mol CO_2_ fixed in the first case vs. 0.05 mol CO_2_ fixed in the second one. This demonstrated that the flushing time of nitrogen gas could affect the fixation of CO_2_. Shorter flushing time, more CO_2_ would be fixed into Na_2_CO_3_. Unfortunately, a large amount of Na_2_CO_3_ in the fermentation broths poses a significant challenge for the separation of 1,3-PDO. Indeed, it is not ignored that a large amount of exhaust gas composed of CO_2_ and H_2_ from fermentation is discharged into the atmosphere. Therefore, a more significant reduction in CO_2_ emissions and soluble salts in the fermentation broth is essential for the industrial fermentation of 1,3-PDO.

#### High 1,3-PDO productivity using Ca(OH)_2_ instead of NaOH

To verify the feasibility of 1,3-PDO and other organic acids production using Ca(OH)_2_ as a CO_2_ capture agent and pH regulator, fed-batch fermentations were performed with different concentrations of Ca(OH)_2_ suspension in comparison to 5 M NaOH solution, as shown in Table 2. In addition, the effect of different stirring speeds (150, 250, 350 rpm) on fermentation was investigated. When 5 M Ca(OH)_2_ suspension was used, the concentration and productivity of 1,3-PDO were higher, i.e., 85.1 vs. 88.6 g/L and 2.84 vs. 5.54 g/L/h, respectively, when compared to the fermentation using 5 M NaOH, as shown in Fig. 1c and Table 2. As the concentration of Ca(OH)_2_ suspension decreased, the concentration and productivity of 1,3-PDO were also reduced. For example, only 76.3 g/L 1,3-PDO was produced if Ca(OH)_2_ concentration decreased to 1.5 M, which is mainly attributed to a dilution effect of low alkaline concentration on fermentation broth. The concentration of butyric acid and acetic acid dropped moderately as a decline of Ca(OH)_2_ concentration. Unexpectedly, lactic acid was generated using Ca(OH)_2_ about twice higher than NaOH as listed in Table 2. The above results led to the reduced yield of 1,3-PDO (0.481–0.486 g/g) in Ca(OH)_2_ group. On the other hand, stirring speed between 150 and 350 rpm had little effect on fermentation, indicating adequate mixture homogeneity of the fermentation broth obtained at the given stirring speeds and no other effects on microbial metabolism [52].

**Table 2:** Fed-batch fermentation using different CO_2_ capture agent and pH regulator

pH regulator	Fermentation period (h)	1,3-PDO (g/L)	Butyrate (g/L)	Acetate (g/L)	Lactate (g/L)	Q_1,3-PDO_ (g/L/h)	Yield (g _1,3-PDO_/g gly)
5M NaOH^a^	30	85.1 ± 1.2	14.5 ± 0.2	9.63 ± 0.1	1.32 ± 0.0	2.84	0.504
5M Ca(OH)_2_^a^	16	88.6 ± 0.3	15.7 ± 0.1	9.42 ± 0.1	2.91 ± 0.1	5.52	0.486
2.5M Ca(OH)_2_^a^	16	81.1 ± 1.1	15.0 ± 0.4	8.63 ± 0.1	2.55 ± 0.1	5.14	0.481
1.5M Ca(OH)_2_^a^	19	76.3 ± 1.3	14.2 ± 0.4	8.15 ± 0.0	2.21 ± 0.0	4.09	0.483
5M Ca(OH)_2_^b^	16	88.3 ± 0.5	15.8 ± 0.5	9.34 ± 0.2	3.03 ± 0.1	5.51	0.485
5M Ca(OH)_2_^c^	16	84.5 ± 0.9	15.2 ± 0.4	9.01 ± 0.3	2.82 ± 0.0	5.28	0.483
NaOH & Ca(OH)_2_^a^	17	84.4 ± 0.6	14.2 ± 0.3	9.81 ± 0.1	1.17 ± 0.0	4.96	0.506

Stirring speed: ^a^250 rpm; ^b^350 rpm; ^c^150 rpm

Up to date, the highest concentration of 1,3-PDO produced by natural producers was 104.8 g/L with a productivity of 3.38 g/L/h, which was achieved in fed-batch fermentation using pure glycerol and a large amount of yeast extract in our previous study [34]. In continuous fermentation, the highest 1,3-PDO productivity was 13.3 g/L/h with a lower 1,3-PDO concentration of 26.5 g/L [53]. The productivity of 1,3-PDO would decrease to 5.55 g/L/h if the 1,3-PDO concentration increased to 57.86 g/L [32]. In this study, the productivity of 1,3-PDO (5.54 g/L/h) obtained in fed-batch fermentation with 5 M Ca(OH)_2_ suspension was surprisingly 31.9% higher than the previously reported highest level (4.20 g/L/h) of fed-batch fermentation and reached the productivity of continuous fermentation [32, 54]. Moreover, the concentration of 1,3-PDO (88.6 g/L) in fed-batch fermentation was much higher than in continuous fermentation (57.86 g/L) with comparable productivity [32].

It should be emphasized that such high productivity and concentration were achieved in a much shorter fermentation period (16 h) using Ca(OH)_2_ than NaOH (30 h). A comparison of Fig. 1a, c illustrated that a rapid accumulation of 1,3-PDO occurred from the same start of 8.00 g/L at 3 h to the different end of 64.0 g/L at 13 h in Fig. 1a or 76.1 g/L at 11 h in Fig. 1c, resulting in productivities of 5.60 and 8.51 g/L/h, respectively. In addition, the production trend of 1,3-PDO was not weakened until the end of fermentation in the 5 M Ca(OH)_2_ group, whereas a significant reduction occurred at the end of fermentation in the 5 M NaOH group. Despite the fact that adding a larger amount of Ca(OH)_2_ suspension dilutes the fermentation broth, the total 1,3-PDO concentration and productivity achieved in the Ca(OH)_2_ group were higher than in the NaOH group. This might be due to both Ca^2+^ stimulation on cell growth and a decrease in osmotic pressure, which is relevant to adding Ca(OH)_2_ during fermentation. Undoubtedly, a high osmotic pressure caused by high soluble salt concentration, e.g., NaOH group, poses a challenge to cell survival and metabolism [55]. The desalination of Ca(OH)_2_ group will be discussed in Sect. Desalination and deprotein of the fermentation broth. As coupling by-products, butyric acid and acetic acid were found to be positively correlated with the production of 1,3-PDO. The comparative analysis demonstrated that a considerable amount of lactic acid was produced in Ca(OH)_2_ group compared to the 5 M NaOH group. It was reported that the presence of Ca^2+^ could contribute to the formation of lactic acid [56, 57]. In addition, lactic acid production might be related to citric acid and PO_4_^3−^ in the fermentation, which will be discussed in detail in Sect. Desalination and deprotein of the fermentation broth

Although the yield of 1,3-PDO (0.481–0.486 g/g) was reduced in the Ca(OH)_2_ group, it was comparable to the reported yield of *K. peneumoniae*. For example, a yield of 0.454 g /g with 80.2 g/L 1,3-PDO was obtained by *K. peneumoniae* DSM4799 from crude glycerol [38], and *K. peneumoniae* LX3 obtained a yield of 0.487 g/g accompanied by 71.4 g/L 1,3-PDO using refined glycerol as the substrate [58]. In contrast to *K. peneumoniae*, the production of 1,3-PDO by *C. butyricum* DL07 was still competitive using Ca(OH)_2_ in the fermentation because of the high concentration of 1,3-PDO (88.6 g/L) with a considerable productivity (5.54 g/L/h) from crude glycerol. Moreover, the CO_2_ produced in the fermentation was captured in large quantities, resulting in the insoluble CaCO_3_ in the fermentation broths. This process is environment-friendly while relieving the pressure of 1,3-PDO separation. Specific details will be discussed later.

#### Improvement of 1,3-PDO yield using double CO_2_ capture agents

A two-stage pH control strategy using double pH regulators has been explored to produce docosahexaenoic acid [59]. The combined application of various CO_2_ capture agents was attempted for the production of 1,3-PDO, aiming at increasing the 1,3-PDO yield and reducing the soluble salt concentrations in the fermentation broth. In the first 12 h of fermentation, 5 M NaOH was used to capture CO_2_ as well as to regulate the fermentation pH, and then 5 M Ca(OH)_2_ suspension instead of 5 M NaOH was employed to proceed with the fermentation. The results showed that the concentration of 1,3-PDO was 84.4 g/L in 17 h, accompanied by a productivity of 4.96 g/L/h (Fig. 1d). More importantly, the yield of 1,3-PDO reached 0.506 g 1,3-PDO /g glycerol, which was equal to that of the 5 M NaOH group. It is reported that the maximum theoretical 1,3-PDO yield is 0.72 mol 1,3-PDO/mol glycerol (about 0.60 g 1,3-PDO/g glycerol), which is calculated by kinetic analysis of *C. butyricum* based on no formation of hydrogen and butyric acid [60]. Regardless of CO_2_ capture agent and pH regulator (NaOH, Ca(OH)_2_ or NaOH & Ca(OH)_2_), *C. butyricum* DL07 could produce up to 84.4–88.6 g/L 1,3-PDO with the yield of 0.481–0.506 g 1,3-PDO/g glycerol. Moreover, the 1,3-PDO productivity was about 2.84–5.54 g/L/h. These values indicated that *C. butyricum* DL07 had excellent 1,3-PDO production performance according to 1,3-PDO production using different pH regulators in some recent reports (Table 3). Double CO_2_ capture agents prevented the reduction of yield of 1,3-PDO in the Ca(OH)_2_ group, while butyric acid (14.2 g/L) and acetic acid (9.81 g/L) showed negligible changes. The production of lactic acid was only 1.17 g/L, which was similar to that of NaOH group, but greatly different from the Ca(OH)_2_ group (Additional file 1: Fig. S1). Little difference in the production of lactic acid within 5 h of the fermentation occurred in the three groups. However, after 5 h of fermentation, lactic acid concentration continued to rise in the Ca(OH)_2_ group, but remained almost unchanged in both the NaOH group and the double alkalis group (NaOH & Ca(OH)_2_).

**Table 3:** 1,3-PDO production by natural 1,3-PDO producers from glycerol in fed-batch fermentation

Microorganism	Titer (g/L)	Yield (g/g)	Overall productivity (g/L/h)	pH regulator	Refs.
*K. pneumoniae* HSL4	80.1	0.44	2.22	NaOH	[61]
*K. pneumoniae* LX3	71.4	0.49	2.24	NaOH	[58]
*K. pneumoniae* KXJPD-Li	65.3	0.46	1.36	KOH	[62]
*C. butyricum* VPI 1718	67.9	0.55	0.78	NaOH	[35]
*C. butyricum* AKR 102	50.5	0.47	1.80	NaOH	[28]
*C. butyricum* SCUT343-4	59.2	0.53	2.11	NaOH	[54]
*C. butyricum* (Gen 7)	66.2	0.51	1.38	NaOH	[63]
*C. pasteurianum*	81.2	0.49	4.27	Ammonia	[64]
*C. freundi*i FMCC-B294	68.1	0.40	0.79	NaOH	[65]
*C. freundi*i VK19	47.2	0.38	0.73	NaOH	[66]
^a^*L. reuteri* DSM 20016	52.3	0.51	1.09	Ammonia	[67]
Mixed culture	70.0	0.56	2.60	NaOH	[68]
Microbial consortium C2-2 M	82.7	0.54	3.06	NaOH	[69]
Microbial consortium CJD-S	41.5	0.34	1.15	NaOH	[70]
*C. butyricum* DL07	85.1	0.50	2.84	NaOH	This study
	88.6	0.49	5.54	Ca(OH)_2_	
	84.4	0.51	4.96	NaOH & Ca(OH)_2_	

^a^Glycerol and xylose as co-substrate

In the process of converting glycerol to 1,3-PDO by *C. butyricum* DL07, when Ca(OH)_2_ was the only CO_2_ capture agent and pH regulator, the productivity of 1,3-PDO was greatly improved. Regrettably, the yield of 1,3-PDO has decreased. A process with satisfactory 1,3-PDO yield, productivity and concentration is expected to reduce 1,3-PDO production costs. Fortunately, using double CO_2_ capture agents resulted in an increase in 1,3-PDO yield (0.506 g/g) with satisfactory 1,3-PDO concentration (84.4 g/L) and productivity (4.96 g/L/h). Furthermore, the production of lactic acid showed the same trend (almost constant concentration from 5 h to the end point of fermentation) in the double CO_2_ capture agents group as compared to the NaOH group, whereas more lactic acid was produced after 5 h of fermentation in Ca(OH)_2_ group (Additional file 1: Fig. S1). A large amount of Ca^2+^ has been reported to contribute to the formation of lactic acid [56, 57]. It can be inferred that the presence of a large amount of Ca^2+^ at the early stage of fermentation could activate metabolic pathways of lactic acid, resulting in a continuous increase of lactic acid production until the end of fermentation and a further reduction in 1,3-PDO yield (Additional file 1: Fig. S1). When NaOH was used rather than Ca(OH)_2_ in the early fermentation stage, that is, there is little Ca^2+^ in the early fermentation stage, and lactic acid was not produced in large quantities. As a result, the 1,3-PDO yield was improved using double CO_2_ capture agents. It is a promising process to employ double CO_2_ capture agents and pH regulators in fermentation to achieve high concentration, yield, and productivity of 1,3-PDO.

### In situ carbon dioxide capture to form micro-nano calcium carbonate

**Fig. 2:**
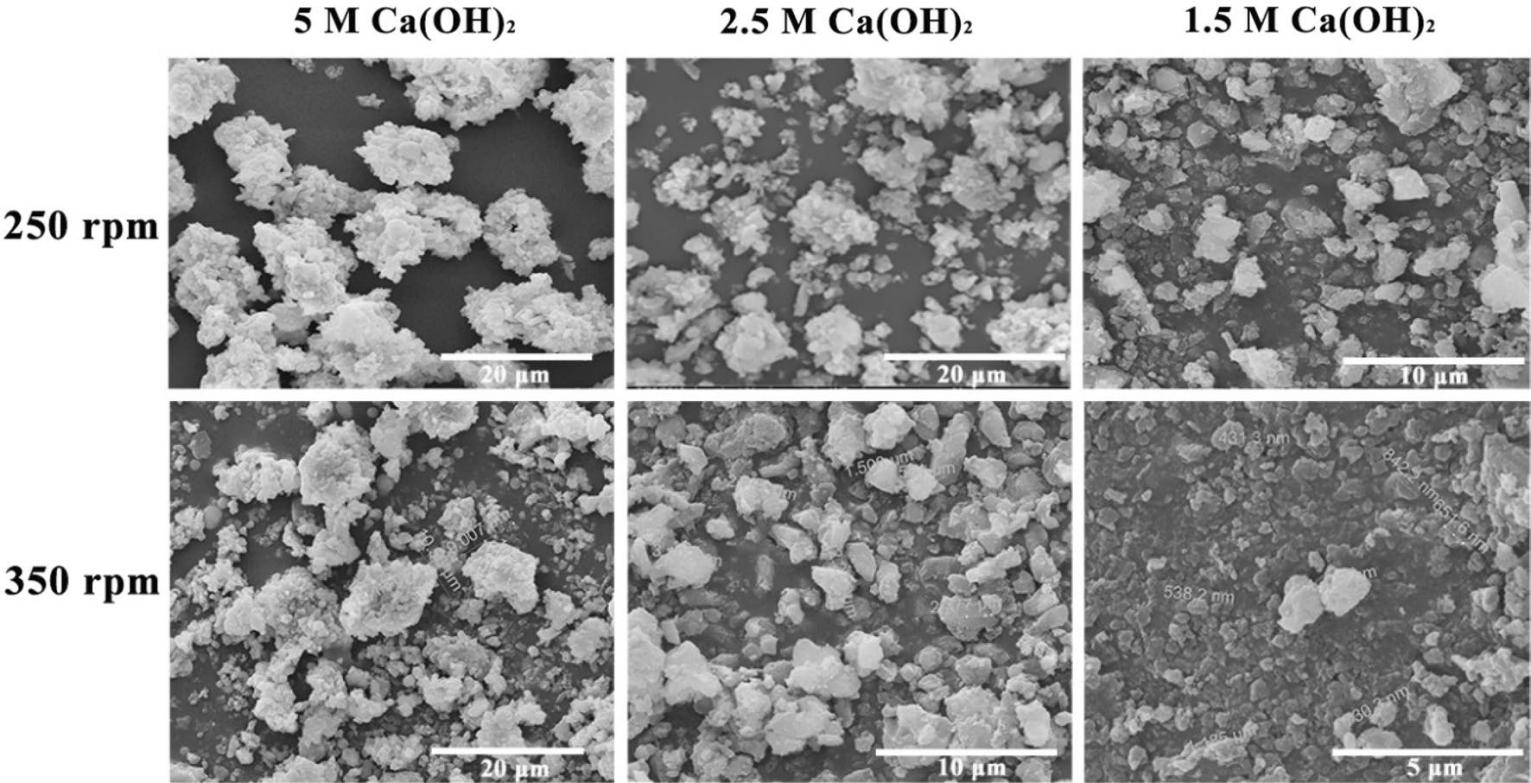
SEM images of precipitations produced during fermentation at different Ca(OH)_2_ concentrations and stirring speeds

H_2_ and CO_2_ as exhaust gases are produced in glycerol metabolism by *C. butyricum*. In fermentation, Ca(OH)_2_ acts as a CO_2_ capture agent allowing for cleaner production and the synthesis of valuable products. In theory, CaCO_3_ could be generated in fermentation once Ca(OH)_2_ reacts with CO_2_. The concentration of Ca(OH)_2_ suspension and the stirring speed of fermentation have a non-negligible effect on the size of CaCO_3_ particles [44]. As a result, the production of CaCO_3_ was investigated using various Ca(OH)_2_ concentrations and fermentation stirring speeds (Fig. 2). When 5 M Ca(OH)_2_ suspension was used as a CO_2_ capture agent at the stirring speed of 250 rpm, the highest amount of precipitate (76.8 g/L) was obtained in fermentation. The precipitates showed a decreasing trend as the Ca(OH)_2_ concentration was reduced. Subsequently, the precipitations collected from fermentations were observed by SEM. Most particles with a size of 10–15 μm were produced using 5 M Ca(OH)_2_ suspension and the stirring speed of 250 rpm. When the concentration of Ca(OH)_2_ suspension was reduced to 2.5 M, a large proportion of particles were distributed between 5 and 10 μm. Using 1.5 M Ca(OH)_2_ suspension as the CO_2_ capture agent, particles with a large size limit of 5 μm were obtained. In addition, by increasing the fermentation stirring speed to 350 rpm, further small-size precipitations were observed. Precipitations in fermentation were smaller than 5 μm in the 2.5 M Ca(OH)_2_ group. Surprisingly, the nano-particles were produced using 1.5 M Ca(OH)_2_ suspension. The precipitations were not completely individual particles, according to the SEM images, but a portion of them were aggregated.

**Fig. 3:**
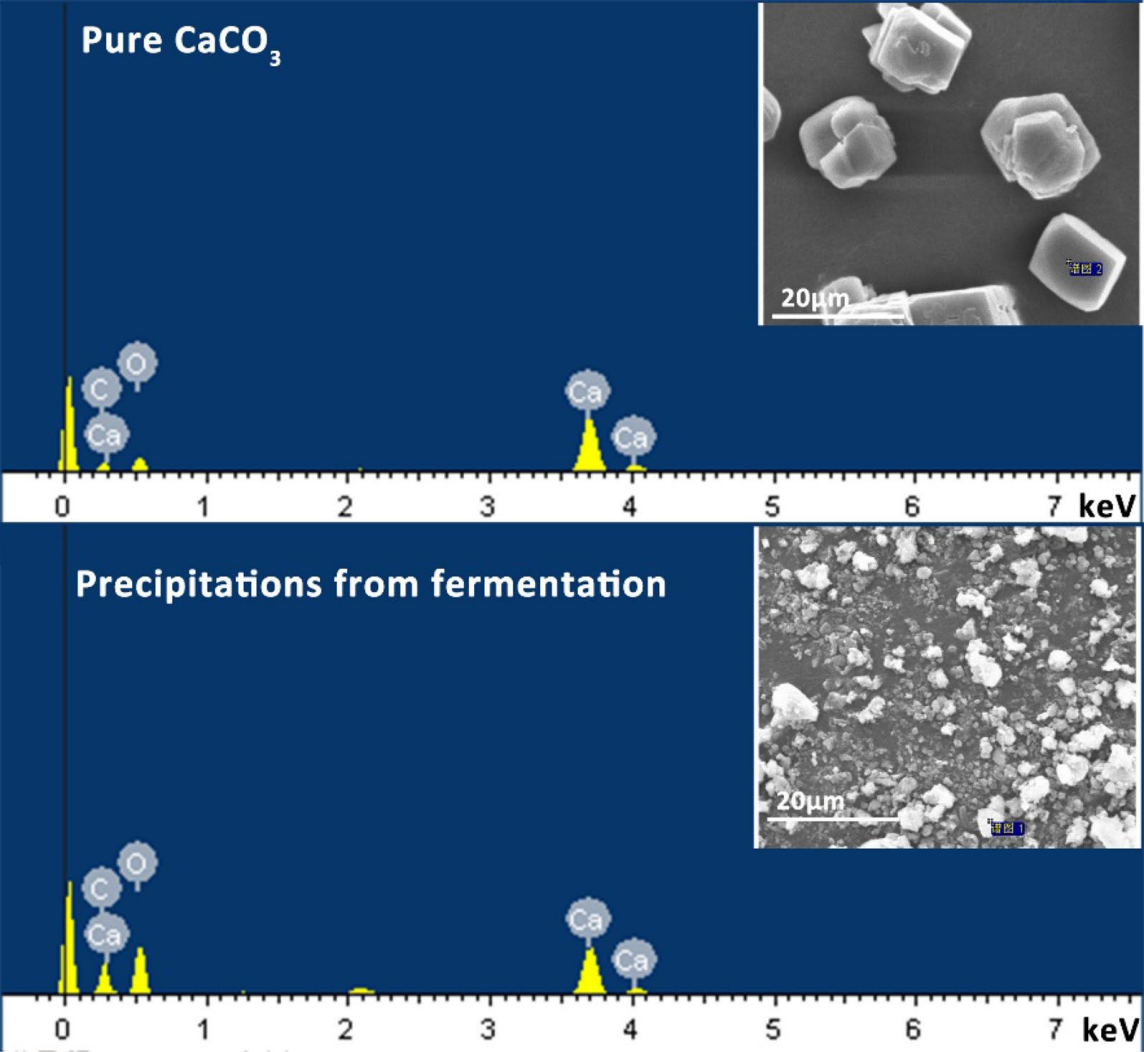
EDS and SEM images of pure CaCO_3_ and precipitations collected in the fermentation

Elemental composition analysis was performed by energy dispersive X-ray spectroscopy (EDX) at 15.0 keV to identify the elemental compositions in the precipitations. The EDX spectra for the precipitations in 5 M Ca(OH)_2_ group was presented in Fig. 3. Moreover, the EDX analysis of commercial pure CaCO_3_ was also conducted to serve as standard sample. As a result, calcium, carbon, and oxygen as the most abundant elements were determined in precipitations collected in the fermentation. Further comparison with the EDX spectra of pure CaCO_3_, revealed a high degree of similarity in the EDX spectra between these precipitations and pure CaCO_3_. it can be concluded that the precipitations produced during fermentation were CaCO_3_ particles. Finally, the crystal form of CaCO_3_ particles was characterized by XRD. As indicated by XRD spectra (Additional file 1: Fig. S2), the intensive peaks of the sample exhibited high agreement with the standard spectrum (Calcite CaCO3: PDF#86-2334), demonstrating that the generated CaCO_3_ particles existed in calcite crystal.

It has been reported that many microorganisms, including *Bacillus pasteurii, Bacillus mucilaginosus, Bacillus alcalophilus, Photosynthetic bacteria* could induce CaCO_3_ formation except *C. butyricum* [46, 71, 72]. The CO_2_ released by *C. butyricum* could undoubtedly react with Ca(OH)_2_, resulting in the formation of CaCO_3_. The precipitations of various sizes in the fermentation were observed by a SEM, while the concentration of Ca(OH)_2_ and the stirring speed of the fermentation were changed. Obviously, the size of CaCO_3_ particles formed in fermentation decreased as the concentration of Ca(OH)_2_ suspension decreased and the stirring speed of fermentation increased. After all, the stirring speed can affect the precipitation crystal and size [73, 74]. The synthesis of micro-particles was accomplished under 2.5–5 M Ca(OH)_2_ suspension with 250–350 rpm stirring speed as well as 1.5M Ca(OH)_2_ suspension with 250 rpm stirring speed. The nano-particles were synthesized using the 1.5 M Ca(OH)_2_ suspension and a stirring speed of 350 rpm. Therefore, it can be concluded that micro/nano CaCO_3_ was produced as calcite during the production of 1,3-PDO by *C. butyricum* DL07. Bacteria could induce various crystalline CaCO_3_ in the form of calcite, aragonite, vaterite and amorphous CaCO_3_. In general, the effects of induction conditions and time on the crystal form and size of CaCO_3_ are investigated using microbial induction method, but the productivity of CaCO_3_ is rarely reported. Magnesium, potassium, phosphate, citric acid, amino acids, polysaccharides, and proteins have all been linked to the formation of CaCO_3_ crystals [45–48]. It can be considered that the formation of calcite was related to the presence of magnesium, potassium, phosphate, citric acid, glycerol and so on in fermentation broth, and even to the polysaccharides and proteins produced by *C. butyricum* DL07. A complex process resulted in the production of stable calcite (CaCO_3_), allowing CO_2_ to be converted into high value products during the fermentation. Although calcite is the most common crystalline form, the time required to form CaCO_3_ was reduced from more than 3 days reported by microbial induced method to 16 h using glycerol fermentation [46]. To our knowledge, micro-nano CaCO_3_ and *C. butyricum* could be obtained separately by differential centrifugation after fermentation because of their different sedimentation coefficients. Calcite CaCO_3_ could be widely applied in the construction industry. Moreover, a popular mixture of CaCO_3_ and living probiotics can be widely used as animal feed.

### Improved ratio of hydrogen in the exhaust gas

**Fig. 4:**
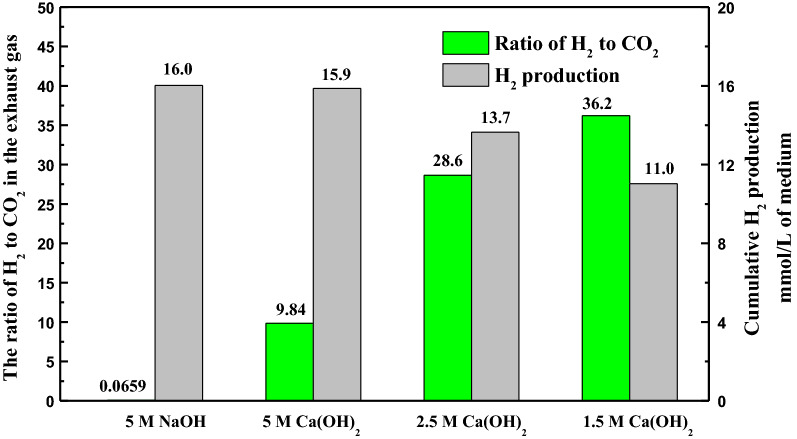
Ratio of H_2_ to CO_2_ and H_2_ production in the fermentation with different concentrations of CO_2_ capture agent

In general, a mixture of H_2_ and CO_2_ as exhaust gas was released from most microbial fermentations into the environment [75, 76]. Based on Ca(OH)_2_ as CO_2_ capture agent, the composition of exhaust gas was detected and expressed as percentage difference (Fig. 4), with 5 M NaOH as the control. At the end of fermentation, the ratio of H_2_ to CO_2_ in exhaust gas was 0.0659 in the NaOH group, accounting for a CO_2_ composition of 92.4% along with 6.09% H_2_ (Additional file 1: Fig. S3). When Ca(OH)_2_ was selected as a CO_2_ capture agent, the ratio of H_2_ to CO_2_ increased significantly, i.e., 9.84 with a H_2_ proportion of 17.2% in the 5 M Ca(OH)_2_ group, 28.6 in the 2.5 M Ca(OH)_2_ group, and 36.2 in the 1.5 M Ca(OH)_2_ group. Regrettably, in the double CO_2_ capture agents group, the ratio of H_2_ to CO_2_ was reduced to 0.203, with a H_2_ proportion of 9.21% and CO_2_ proportion of 45.3% in exhaust gas. The highest H_2_ production of 15.9 mmol/L of medium was achieved using 5 M Ca(OH)_2_ suspension and 11.0 mmol/L of medium as the lowest concentration was produced with 1.5 M Ca(OH)_2_ suspension as the CO_2_ capture agent.

*C. butyricum* is commonly employed to produce H_2_ [77]. The ratio of H_2_ to CO_2_ in exhaust gas of fermentation would increase if CO_2_ was captured, which will contribute to the separation of H_2_. Expectedly, the proportion of CO_2_ significantly decreased, because CO_2_ was converted into CaCO_3_ particles in the fermentation. Indeed, there was a significant reduction in the proportion of CO_2_ (< 1.75%) in the 5 M Ca(OH)_2_ group. The results showed that the ratio of H_2_ to CO_2_ was 152 times higher in the 5 M Ca(OH)_2_ group than the 5 M NaOH group indicating that Ca(OH)_2_ had a more outstanding CO_2_ fixation level. As the Ca(OH)_2_ concentration decreased, the production of H_2_ and CO_2_ was declined, but has led to a rising ratio of H_2_ to CO_2_ (Fig. 4 and Additional file 1: Fig. S3). This indicated that a reduced productivity of CO_2_ in fermentation caused by lower Ca(OH)_2_ concentration made Ca(OH)_2_ to fix CO_2_ more efficiently. Thus, the proportion of CO_2_ in the exhaust gas decreased as Ca(OH)_2_ concentration decreased, and the value was almost close to zero. On the contrary, the proportion of H_2_ in the exhaust gas increased slightly as Ca(OH)_2_ concentration decreased. Therefore, the H_2_/CO_2_ ratio decreased with Ca(OH)_2_ concentration significantly. Apparently, the ratio of H_2_ to CO_2_ in the double CO_2_ capture agents group (0.203) had a significant decrease compared to the Ca(OH)_2_ group due to the poor CO_2_ capture agent (NaOH) acting in the first 12 h of the fermentation. There is no doubt that more CO_2_ would be fixed to form CaCO_3_ in situ as long as the excellent CO_2_ capture agent (Ca(OH)_2_) was employed as soon as possible in the fermentation, surely obtaining a higher ratio of H_2_ to CO_2_. Therefore, it can be concluded that the earlier Ca(OH)_2_ is introduced into fermentation, the higher H_2_ ratio with lower CO_2_ ratio appears in the exhaust gas. In the future, the addition time of Ca(OH)_2_ should be optimized for a higher ratio of H_2_ to CO_2_ in double CO_2_ capture agents. The accumulated H_2_ production ranged between 11.0 and 15.9 mmol/L of medium, which was in agreement with the previous reported [78]. H_2_ production decreased as Ca(OH)_2_ concentration decreased, following the same trend as the production of 1,3-PDO, owing to a dilution effect of Ca(OH)_2_ suspension. Studies on microbial fermentation accompanied by in situ CO_2_ capture are rarely reported. Many studies had reported biohydrogen production using *C. butyricum*, but only focusing on the total hydrogen production, not on the ratio of H_2_ in the exhaust [78–81]. Actually, the ratio of H_2_ to CO_2_ is concerned with the separation of H_2_, since normally CO_2_ plays an indispensable role in most microbial fermentation [49]. This new process proposed in this study provides a reference for microbial fermentation to capture CO_2_ and improves the ratio of H_2_ in the exhaust gas in the future.

### Desalination and deprotein of the fermentation broth

**Fig. 5:**
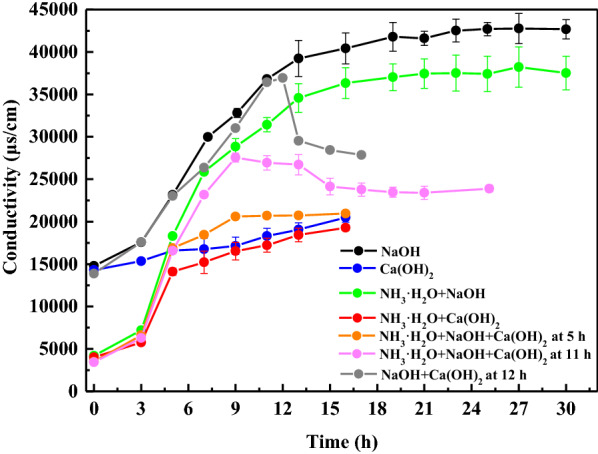
Conductivities of fermentation broths under different CO_2_ capture scenarios. NaOH represents 5 M NaOH solution; Ca(OH)_2_ represents 5 M Ca(OH)_2_ suspension; In the ammonium hydroxide group, ammonium hydroxide acted as an inorganic nitrogen source and, otherwise, (NH_4_)_2_SO_4_ acted as an inorganic nitrogen source

The higher salt concentration in the fermentation broth could affect 1,3-PDO fermentation efficiency, because the high osmotic pressure caused by a large amount of soluble salts poses a challenge to cell survival [82]. More importantly, the high salt concentration can complicate the product separation process [83]. CO_3_^2+^ was present in the fermentation broth, as CO_2_ produced by microbial metabolism dissolved in water. As a result, once Ca(OH)_2_ was selected as the CO_2_ capture agent rather than NaOH, insoluble CaCO_3_ instead of soluble Na_2_CO_3_ would present in the fermentation broth. To further reduce soluble salts in the fermentation broths, quantitative ammonium hydroxide as a pH regulator was added after fermentation initiation to substitute ammonium sulfate of the fermentation media as an inorganic nitrogen source without affecting the 1,3-PDO concentration (Additional file 1: Fig. S4). As expected, differences in conductivity values were observed under different fermentation conditions (Fig. 5). The highest conductivity value of fermentation broth (42,665 μs/cm) was present in the control group (5 M NaOH group). Conversely, the ammonium hydroxide and the 5 M Ca(OH)_2_ group had the lowest conductivity values (19,800 μs/cm). Thus, the salt concentration of the fermentation broth was reduced by 53.6%. The fermentation broth showed a relatively low conductivity when ammonium hydroxide replaced ammonium sulfate of the fermentation media, mainly due to reduced sulfate. Apparently, when Ca(OH)_2_ was added into the fermentation, the conductivity values rose slowly during the subsequent fermentation, indicating the production of insoluble salts. It demonstrated that the addition of Ca(OH)_2_ can effectively reduce the soluble salt concentrations of the fermentation broth, thereby contributing to the separation of 1,3-PDO. In addition, the productivity and concentration of 1,3-PDO in Ca(OH)_2_ group were significantly higher than in NaOH group. It is quite possible that the formation of insoluble CaCO_3_ would result in lower osmotic pressure of fermentation broth, which leads to a lower osmotic pressure for cell, which is beneficial for cell survival and 1,3-PDO production.

For using double CO_2_ capture agents, when 5 M Ca(OH)_2_ suspension was introduced in fermentation at 5 h, the conductivity value of the fermentation broth was 20,960 μs/cm, whereas when Ca(OH)_2_ was involved in fermentation from 11 h, the conductivity was 23,900 μs/cm. Thus, the earlier Ca(OH)_2_ was involved in fermentation, the less soluble salts were present in the fermentation broth. To further clarify the main ion species and specific concentration existed in the fermentation broths, concentration of ions was determined at the end of fermentation. The results showed that large differences in concentration were exhibited in some ions (SO_4_^2^, PO_4_^3−^, Na^+^, Ca^2+^) during the fermentation with different CO_2_ capture agents (Table 4). Compared to the 5 M NaOH group (13,043.60 mg/L), the concentration of Na^+^ of fermentation broth was reduced by 99.7% in the 5 M Ca(OH)_2_ group (38.61 mg/L). Conversely, Ca^2+^ concentration was increased from 64.74 mg/L to 6308.75 mg/L. When Ca(OH)_2_ participated in the fermentation, there was no PO_4_^3−^ in the fermentation broth. This indicated that Ca_3_(PO_4_)_2_ precipitations existed in the fermentation broth. At the same time, regardless of the fermentation conditions, the NH_4_^+^ added to the media was almost absent at the end of the fermentation, which should be attributed to the microbial uptake. There was little difference in the concentration of Cl^−^ and K^+^ concentrations, although different CO_2_ capture agents were used in the fermentation. Overall, the total ion concentration in Ca(OH)_2_ group was about half that of the NaOH group.

**Table 4:** Ions concentration of fermentation broth using different CO_2_ capture agents and pH regulators

Ions (mg/L)	A	B	C	D
Cl^−^	221.65	169.50	190.40	171.42
SO_4_^2−^	3268.58	539.96	14.28	3.57
PO_4_^3−^	423.68	0.00	403.42	0.00
Na^+^	13,043.60	38.61	10,865.96	47.55
K^+^	453.91	446.88	454.60	451.85
NH_4_^+^	0.00	0.00	3.62	5.91
Ca^2+^	64.74	6308.75	90.00	5658.55

A and C represent 5 M NaOH as the CO_2_ capture agent and pH regulator; B and D represent 5 M Ca(OH)_2_ as the CO_2_ capture agent and pH regulator; (NH_4_)_2_ SO_4_ was supplemented into the medium of A and C; Ammonium hydroxide was added in B and D group during the fermentation

Surprisingly, there were also significant differences in soluble protein concentration of fermentation broth when 5 M Ca(OH)_2_ suspension and 5 M NaOH were selected as the CO_2_ capture agent and pH regulator, respectively. The soluble protein concentration of 5M Ca(OH)_2_ group was only 1.52 g/L, while that of the 5 M NaOH group was up to 2.72 g/L. This result suggested that the soluble proteins of the fermentation broth decreased by 44.1% with 5 M Ca(OH)_2_ suspension as the CO_2_ capture agent and pH regulator when compared to 5 M NaOH. It is speculated that the reduced salt concentrations in fermentation broth caused by insoluble CaCO_3_ pose fewer threats to cells, allowing for the avoidance of proteins synthesis and bacterial cell lyses. After all, high osmotic stress imposes pressure on bacteria to synthesize multiple characteristic proteins, such as membrane proteins and translocators [84], and it may even regulate bacterial death to release intracellular proteins [85]. Lower soluble salt and protein concentrations can effectively facilitate the separation of 1,3-PDO while alleviating the wastewater treatment problem, thereby contributing to sustainable development goals.

## Conclusions

Higher concentrations of 1,3-PDO (> 80.0 g/L) were accomplished from waste crude glycerol by *C. butyricum* DL07 either using NaOH, Ca(OH)_2_ or both combinations as the CO_2_ capture agent and pH regulator. The highest 1,3-PDO concentration (88.6 g/L) accompanied by the highest reported productivity (5.54 g/L/h) was achieved, while 5 M Ca(OH)_2_ suspension acted as the CO_2_ capture agent in fed-batch fermentation. Simultaneously, CO_2_ was captured in situ to produce CaCO_3_ and improve the ratio of H_2_ to CO_2_ in the exhaust gas. Furthermore, the CaCO_3_ produced during fermentation existed in calcite, and its size gradually decreased as the concentration of the Ca(OH)_2_ suspension decreased and fermentation speed increased. Nano-CaCO_3_ was synthesized in 1.5 M Ca(OH)_2_ group with a stirring speed of 350 rpm. More importantly, the produced CO_2_ by waste crude glycerol fermentation was almost entirely captured in situ, which resulted in a 152-fold increase in the ratio of H_2_ to CO_2_. In this process, the conductivity of the fermentation broth was reduced significantly in Ca(OH)_2_ group because of the generation of insoluble CaCO_3_. When ammonium hydroxide was added in the 5 M Ca(OH)_2_ group as an inorganic nitrogen source instead of (NH_4_)_2_SO_4_, the conductivity of the fermentation broth was reduced by 54.3% when compared to the 5 M NaOH group. The soluble protein concentration of fermentation broth was obviously decreased by 44.1%, while 5 M Ca(OH)_2_ suspension was employed as the CO_2_ capture agent and pH regulator rather than 5 M NaOH. The reduced soluble salt and protein concentrations in the fermentation broth will significantly contribute to the downstream product separation process. Undoubtedly, the pressure of wastewater treatment would also be relieved, promoting the green production process. This new process integrates the generation of important chemicals (1,3-PDO), material (CaCO_3_) and clean energy (H_2_) using waste glycerol with a dramatically reduced carbon footprint. Therefore, this process provides a green and environmentally friendly production method for 1,3-PDO, which is highly consistent with sustainable development goals and serves as a valid reference for the production of bio-based chemicals to achieve net-zero CO_2_ emissions. It will broaden the range of applications for bio-based chemicals.

## Methods

### Microorganism and culture media

*C. butyricum* DL07, previously selected from anaerobic active sludge [34], was used in this study. It was stored at − 70 °C using a seed medium with 40% glycerol in the lab as well as China General Microbiological Culture Collection Center (CGMCC NO. 17934). The seed and fermentation media were prepared as described in our previous work [34], but ammonium sulfate was not added to the fermentation medium when ammonia was involved in controlling the fermentation pH. Crude glycerol was used as substrate in seed and fermentation media. 100 mL of seed medium was fed into 250 mL anaerobic serum bottles and bubbled with nitrogen gas. The seed and fermentation media were sterilized at 121 °C for 20 min. Crude glycerol was supplied from Sichuan Tianyu Oleochemical Co. Ltd., China. Its components had been described in previous study [69].

### Culture conditions

*C. butyricum* DL07 was revived and cultured in seed medium. 4% (v/v) of strain suspension was inoculated into the seed medium. The seed culture was carried out in shaker at 37 °C and 200 rpm for 12 h. Fermentations were performed in a 5.0 L bioreactor (Baoxing Biotech, Shanghai, China) containing 2.0 L fermentation medium. To ensure an anaerobic environment, N_2_ was bubbled into the fermentation medium at 0.15 vvm, starting at 1 h before inoculation, and then stopped at 1 h after inoculation. 10% (v/v) of inoculum was inoculated into the bioreactor. The bioreactor was automatically run at various stirring speeds according to the experimental design. Throughout the fermentation process, the pH was maintained by different concentrations of Ca(OH)_2_ suspension involving NaOH and ammonia (6.80 g/L). Ca(OH)_2_ suspension was stirred at 100 rpm during service period.

### Fed-batch fermentations for the co-production 1,3-PDO, CaCO_3_ and H_2_

Fed-batch fermentations were carried out using continuous feeding strategy. The initial glycerol concentration was 40 g/L. When the residual glycerol concentration dropped to 20 g/L, crude glycerol was manually pumped into the bioreactor to maintain the glycerol concentration of about 20 g/L during the fermentation. The detailed operation of fermentation can refer to the previous report [34]. The exhaust gas produced in the bioreactor was collected manually using a 1 L gas collection bag. All CaCO_3_ deposits were collected by centrifugation at 5000 rpm for 10 min.

### Analytical methods

Cell mass was represented by the optical density of the sample at 650 nm using a UV–visible spectroscopy system (UV-5100, Metash Instruments Co. Ltd, Shanghai, China). The concentrations of 1,3-PDO, glycerol and acids (butyric acid, acetic acid and lactic acid) were analyzed by high performance liquid chromatography (Waters, Milford, USA) equipped with an Aminex HPX 87H column (300 × 7.8 mm) ( Bio-Rad, Hercules, CA), a differential refractometer (Waters 2414) and an autosampler (Waters 2707). Operating conditions applied for detector temperatures, column temperature, the flow rate of mobile phase (5 mM H_2_SO_4_) and sample volume were 35 °C, 65 °C, 0.6 mL/min and 20 μL, respectively. Each testing sample from the fermentation broths was centrifuged for 10 min at 12,000 r/min. The clarified fermentation broth and chloroform were mixed at a ratio of 1:1 and centrifuged again under the above conditions to remove soluble proteins. After proper dilution, the above sample was filtered through a 0.22 μm membrane filter for analysis. All the precipitates were washed twice with pure water to remove impurities before being dried at 50 °C until constant weight for the collection of CaCO_3_. The collected exhaust gas was detected by gas chromatography (GC-7900, Techcomp Co., Ltd. Shanghai, China). The conductivity of fermentation broth was measured using an intelligent conductivity meter. The ions in the fermentation broth were analyzed using high-performance ion chromatography. The concentration of soluble proteins contained in the fermentation broth was determined by the bradford protein assay kit (Beyotime Biotech, Shanghai, China).

The fixed CO_2_ (mol) in the fermentation was calculated according to the following formula. Where consumption of NaOH is the consumption of NaOH in the fermentation. Total organic acids are the total organic acids produced in the fermentation.

$${\text{Fixed CO}}_{{2}} \left( {{\text{mol}}} \right) \, = {\text{ Consumption of NaOH }}\left( {{\text{mol}}} \right) \, {-}{\text{ Total organic acids }}\left( {{\text{mol}}} \right)$$

### CaCO_3_ particles characterization

The elemental composition of precipitations was analyzed by energy dispersive spectrometer (EDS) equipped with an SEM instrument. The morphology of CaCO_3_ was examined by a scanning electron microscopy (SEM; FEI Quanta 450, The USA). The polymorphs of CaCO_3_ particles were characterized via X-ray diffraction (XRD) with Rigaku D/max 2400 V diffractometer (Japan).

## Supplementary Information

**Additional file 1**: **Fig. S1 **Production of lactic acid using different CO_2_ capture agents. The solid symbol represents lactic acid production, and the hollow symbol represents citric acid consumption. The stirring speed is 250 rpm in every groups. **Fig. S2** XRD pattern of CaCO_3_ from the fermentation. **Fig. S3** Ratio of H_2_ and CO_2_ in exhaust gas using different CO_2_ capture strategies. **Fig. S4** 1,3-PDO production under different CO_2_ capture scenarios. NaOH represents 5 M NaOH solution; Ca(OH)_2_ represents 5 M Ca(OH)_2_ solution; In the ammonium hydroxide group, ammonium hydroxide acted as an inorganic nitrogen source and, otherwise, (NH_4_)_2_SO_4_ acted as an inorganic nitrogen source.

## Data Availability

The data sets supporting the results reported in this article are included within the article and its Additional files.

## Abbreviations

1,3-PDO: 1,3-Propanediol
LCA: Life cycle assessment
TCA: Techno-economic assessment
DHA: Docosahexaenoic acid
EPA: Eicosapentaenoic acid
PTT: Polytrimethylene terephthalate
MIP: Microbially induced precipitation
EPS: Extracellular polymeric substance. EDX: energy dispersive X-ray spectroscopy
SEM: Scanning electron microscopy
