# Supplementary material for: In situ carbon dioxide capture to co-produce 1,3-propanediol, biohydrogen and micro-nano calcium carbonate from crude glycerol by Clostridium butyricum
Source: Biotechnol Biofuels Bioprod. 2022 Sep 3;15:91. doi: 10.1186/s13068-022-02190-2 (PMC9440576; doi:10.1186/s13068-022-02190-2)
Supplement: Supplementary file 1 — Additional file 1: Fig. S1 Production of lactic acid using different CO2 capture agents. The solid symbol represents lactic acid production, and the hollow symbol represents citric acid consumption. The stirring speed is 250 rpm in every groups. Fig. S2 XRD pattern of CaCO3 from the fermentation. Fig. S3 Ratio of H2 and CO2 in exhaust gas using different CO2 capture strategies. Fig. S4 1,3-PDO production under different CO2 capture scenarios. NaOH represents 5 M NaOH solution; Ca(OH)2 represents 5 M Ca(OH)2 solution; In the ammonium hydroxide group, ammonium hydroxide acted as an inorganic nitrogen source and, otherwise, (NH4)2SO4 acted as an inorganic nitrogen source. [file 13068_2022_2190_MOESM1_ESM.docx]

**Additional file 1**

**Legends of Additional file 1 Figures**

**Fig. S1** The production of lactic acid using different CO_2_ capture agents. The solid symbol represents lactic acid production, and the hollow symbol represents citric acid consumption. The stirring speed is 250 rpm in every groups.

**Fig. S2** XRD pattern of CaCO_3_ from the fermentation

**Fig. S3** The ratio of H_2_ and CO_2_ in exhaust gas using different CO_2_ capture strategies

**Fig. S4** The 1,3-PDO production under different CO_2_ capture scenarios. NaOH represents 5 M NaOH solution; Ca(OH)_2_ represents 5 M Ca(OH)_2_ solution; In the ammonium hydroxide group, ammonium hydroxide acted as an inorganic nitrogen source and, otherwise, (NH_4_)_2_SO_4_ acted as an inorganic nitrogen source

Fig. S1 The production of lactic acid using different CO_2_ capture agents. The solid symbol represents lactic acid production, and the hollow symbol represents citric acid consumption. The stirring speed is 250 rpm in every groups.

Fig. S2 XRD pattern of CaCO_3_ from the fermentation

Fig. S3 The ratio of H_2_ and CO_2_ in exhaust gas using different CO_2_ capture strategies

Fig. S4 The 1,3-PDO production under different CO_2_ capture scenarios. NaOH represents 5 M NaOH solution; Ca(OH)_2_ represents 5 M Ca(OH)_2_ solution; In the ammonium hydroxide group, ammonium hydroxide acted as an inorganic nitrogen source and, otherwise, (NH_4_)_2_SO_4_ acted as an inorganic nitrogen source
